# MRJP1-containing glycoproteins isolated from honey, a novel antibacterial drug candidate with broad spectrum activity against multi-drug resistant clinical isolates

**DOI:** 10.3389/fmicb.2015.00711

**Published:** 2015-07-13

**Authors:** Katrina Brudzynski, Calvin Sjaarda, Robert Lannigan

**Affiliations:** ^1^Department of Drug Discovery and Development DepartmentSt. Catharines, ON, Canada; ^2^Pathology and Laboratory Medicine, Western University and London Health Sciences CentreLondon, ON, Canada

**Keywords:** multi-drug resistant, ESBL, clinical isolates, novel antibacterials, honey glycoproteins, Major Royal Jelly Protein

## Abstract

The emergence of extended- spectrum β-lactamase (ESBL) is the underlying cause of growing antibiotic resistance among Gram-negative bacteria to β-lactam antibiotics. We recently reported the discovery of honey glycoproteins (glps) that exhibited a rapid, concentration-dependent antibacterial activity against both Gram-positive *Bacillus subtilis* and Gram-negative *Escherichia coli* that resembled action of cell wall-active β-lactam drugs. Glps showed sequence identity with the Major Royal Jelly Protein 1 (MRJP1) precursor that harbors three antimicrobial peptides: Jelleins 1, 2, and 4. Here, we used semi-quantitative radial diffusion assay and broth microdilution assay to evaluate susceptibility of a number of multi-drug resistant (MDR) clinical isolates to the MRJP1-contaning honey glycoproteins. The MDR bacterial strains comprised three methicillin-resistant *Staphylococcus aureus* (MRSA), four *Pseudomonas aeruginosa*, two *Klebsiella pneumoniae*, two vancomycin-resistant *Enterococci* (VRE), and five ESBL identified as one *Proteus mirabilis*, three *E. coli*, and one *E. coli* NDM. Their resistance to different classes of antibiotics was confirmed using automated system Vitek 2. MDR isolates differed in their susceptibility to glps with MIC_90_ values ranging from 4.8 μg/ml against *B. subtilis* to 14.4 μg/ml against ESBL *K. pneumoniae, Klebsiella* spp. ESBL and *E. coli* and up to 33 μg/ml against highly resistant strains of *P. aeruginosa*. Glps isolated from different honeys showed a similar ability to overcome bacterial resistance to β-lactams suggesting that (a) their mode of action is distinct from other classes of β-lactams and that (b) the common glps structure was the lead structure responsible for the activity. The results of the current study together with our previous evidence of a rapid bactericidal activity of glps demonstrate that glps possess suitable characteristics to be considered a novel antibacterial drug candidate.

## Introduction

The multi-drug resistant (MDR) bacterial pathogens pose a significant threat to control and treat bacterial infections. Pathogens of particular concern include extended-spectrum β-lactamase-producing (ESBL) Gram-negative Enterobacteriaceae, including *Escherichia coli, Klebsiella* spp., *Pseudomonas* spp. and *Proteus* spp., the main causative agents of nosocomial infections. ESBL arose from increased frequency of mutations in β-lactamase gene in response to continuous exposure of bacteria to new classes of β-lactams. Initially chromosomally encoded TEM and SHV β-lactamases now evolved to new varieties of ESBL enzymes that are predominantly located on large plasmids, often together with genes conferring resistance to other antibiotics such as aminoglycosides or tetracyclines (Paterson and Bonomo, 2005). ESBLs are now documented to be able to hydrolyze of oxyimino-β-lactams, which include cefotaxime, ceftriaxone, ceftazidime, and aztreonam, the main clinically available drugs used to treat severe infections (Paterson and Yu, 1999; Bradford, 2001). Among ESBL are carbapenamases, including serine-β-lactamases (KPC, OXA) (Nordmann et al., 2009) and metallo-β-lactamases (MBLs) such as New Delhi metallo-β-lactamase 1 (NDM-1; Bradford, 2001; Queenan and Bush, 2007). ESBL genes are consecutively expressed and can be transfer to other bacterial species by horizontal gene transfer via mobile elements such as plasmids and transposons (Bradford, 2001; Warnes et al., 2012). These mechanisms of transmission effectively accelerate the spread of MDR bacteria causing nosocomial outbreaks worldwide. Because ESBL-producing strains present often MDR phenotypes, in order to overcome their global resistance, new approaches in drug discovery and designs are needed. These challenges led to re-new interest in natural products as an untapped reservoir for drug discovery (Koehn and Carter, 2005; Molinari, 2009; Hayashi et al., 2013).

Our recent research into nature of antibacterial compounds in honey led to isolation and sequence identification of glycoproteins as compounds chiefly responsible for honey antibacterial activity (Brudzynski and Sjaarda, 2014, 2015). MALDI TOF and electrospray quadrupole time of flight mass spectrometry (ESI-Q-TOF-MS/MS) analysis of glycoproteins (glps) showed sequence identity with the Major Royal Jelly Proteins 1 precursor (MRJP1). We have documented that the full-length glps were able to (a) agglutinate bacterial cells due to a several high-mannose structures attached to protein backbone and (b) were able to induce phenotypic changes, (c) increase bacterial membranes permeability and (d) to cause cell lysis. The time-kill kinetics showed a rapid, >5-log_10_ reduction of viable cells after addition of glps to bacterial cultures (Brudzynski and Sjaarda, 2015).

The MRJP1 has been implicated in several cellular functions in honeybee (Schmitzova et al., 1998; Albert et al., 1999; Kupke et al., 2012; Buttstedt et al., 2014) but it has never been linked before to antibacterial effects of honey. The MRJP1 glycoprotein harbors three antimicrobial peptides, Jelleins (Fontana et al., 2004). Synthetic Jelleins showed antibacterial activity *in vitro* against Gram-positive and Gram-negative bacteria (Fontana et al., 2004).

The glps effects on cell morphology, growth rate and bactericidal activity resembled the action of β-lactam antibiotics on cell envelope (Brudzynski and Sjaarda, 2014, 2015). The full length glps and the β-lactams (penicillins, cephalosporins, carbapenems), polymyxins and glycopeptides (vancomycin and teicoplanin) share the ability to cause perturbation of the bacterial cell wall and the membrane integrity. The above classes of drugs structurally differ between each other and affect the membrane integrity by different mechanisms (Bush et al., 1995; Bush, 2012). The unexpected, novel function of MRJP1-containing glycoproteins, its antibacterial activity, is our very recent finding and the mode of action of glps has yet to be elucidated. To gain some insight into this mechanism, we asked a simple question whether general resistance of bacteria to β-lactams would impede their sensitivity to glps.

The primary goal of this study was to determine whether clinical isolates expressing mutli-MDR phenotypes are sensitive to treatment with honey MRJP1-containing glycoproteins.

## Materials and Methods

### Isolation of Honey Glycoproteins

Glycoproteins were isolated from 25% aqueous honey solutions using the agarose-immobilized Concavalin A spin columns (Glycoprotein Isolation Kit, ConA, Thermo Scientific, Rockford, IL, USA) according to the manufacturer’s manual. Two separate spin columns were usually used to isolated glps from one honey variety. The eluates were combined and lyophilized to dryness. The isolation process was monitored by SDS-PAGE followed by Coomassie blue staining.

### Determination of Protein Concentration

Protein quantification was performed by Bradford methods in the microplate version, using the Bio-Rad Protein Assay (Bio-Rad Laboratories Inc., Hercules, CA, USA) according to the manufacturer instruction.

### SDS-PAGE Electrophoresis (1D)

SDS-PAGE was performed according to the method described by Laemmli (1970). Honey glycoproteins were analyzed by 7.5% gel separation gel with attached 5% stacking gel. 30 μl of a honey solution (50% v/v) or 15 μl of a fraction from ConA chromatography were mixed with the sample buffer, denatured at 100°C for 5 min and loaded on a gel. The electrophoresis was carried out in duplicate at a constant current of 100 Volts using a Mini Protean III electrophoresis cell (Bio-Rad Laboratories, Hercules, CA, USA). After electrophoresis, the gel was stained with Brilliant Blue R-250 (Bio-Rad). The molecular weight of the proteins was determined using molecular weight standards (Fermentas Life Sciences, Fisher Scientific, Ottawa, ON, Canada).

### Two-Dimensional Electrophoresis (2D)

Protein samples were rehydrated in a solution of 7 M urea/2 M thiourea/4% CHAPS/0.5% carrier ampholytes/5 mM tributylphosphine. Each sample was loaded onto a 17 cm pH 3-10 non-linear strip (BioRad) and allowed to passively rehydrate for 18 h at 20°C. IEF strips were focused on a BioRad PROTEAN IEF cell at 10,000 Volts for 100,000 Volts/h.

Following the first dimension the IEF strips were equilibrated.in a solution of 50 mM Tris-HCl pH 8.8/6 M urea/30% glycerol/2% SDS prior to loading the second dimension. At this step the strips were also reduced and alkylated using 1% DTT followed by 2.5% iodoacetamide. Once equilibrated, the strips were loaded on a 10% homogeneous Tris- Glycine gel and run overnight at 50 Volts and 12°C.

### Protein Digestion using the Micromass MassPREP Robotic Protein Handling System

Gels were stained using non-destructive silver stain. Protein plugs were excised from the gel using a coring device with a 2 mm diameter. Gel plugs were de-stained in 15 mM potassium ferricyanide/50 mM sodium thiosulfate and reduced in 10 mM dithiothreitol in 100 mM ammonium bicarbonate for 30 min. Samples were alkylated in 55 mM iodoacetamide in 100 mM ammonium bicarbonate for 20 min, washed in 100 mM ammonium bicarbonate and dehydrated in 100% acetonitrile. The samples were digested with 6 ng/uL trypsin (Promega Sequencing Grade Modified Trypsin) in 50 mM ammonium bicarbonate (25 uL) for 5 h at 37°C. Peptides were extracted from the gel plug with a solution of 30 uL 1% formic acid/2% acetonitrile, and then with 24 uL of 50% acetonitrile follow the first extraction. Pooled extractions were evaporated down to a volume of ∼20 uL using a ThermoSavant Speedvac concentrator model SPD121P.

### MALDI-TOF Mass Spectrometry

Spectra were acquired in positive-ion reflector mode and analyzed on a Voyager DePro (Applied Biosystems Corporation) using Data Explorer Version 5.1 software (Applied Biosystems Corporation). The mass range scanned was 1000–3000 Da. Raw spectra were processed under Data Explorer 5.1 software (Applied Biosystems). Peptide peaks with a signal to noise ratio of greater than 2:1 were selected for a database search. Search parameters were set at trypsin with up to one missed cleavage, fixed carbamidomethyl modification of cysteine and variable oxidation of methionine. Database source was the Mascot search engine using the NCBInr data base at 50 ppm mass accuracy.

### Antibacterial Assay of Glycoproteins by Agar Well-Diffusion Method

The plates were prepared using Mueller-Hinton agar (MHA) poured into layer of a 4 mm deep. The 100 μl of bacterial inoculum (10^7^ CFU/ml) was uniformly spread using a bent-glass rod. Wells were performed with a sterile plastic bore of 3 mm diameter. Each well was filled with 8 μl of sample of glycoproteins (40 μg/ml) prepared in three dilutions; undiluted, 2x and 4x diluted with sterile water. Each plate contained well-filled with ampicillin (1 μg/10 μl or 5 μg/10 μl) as a positive control and BSA (5 μg/10 μl), dissolved in the elution buffer from Con A-chromatography, as a negative control. In addition, the reference MHA plates were produced, inoculated with 100 μl of either *B. subtilis* or *E. coli* (10^7^ CFU/ml) that contained wells filled with series of ampicillin concentrations ranging from 2.5 to 500 μg/ml. The agar plates were incubated for 24 h at 37°C. After incubation, the zone of inhibition of the bacterial growth was measured using a digital, electronic caliper (Flower Company Inc., Newton, MA, USA). Tests were performed in duplicate.

### Broth Microdilution Assay and Determination of the MIC

The susceptibilities of clinical isolates and standard bacteria (10^6^ CFU/ml) to honey glycoproteins were analyzed using broth microdilution assay in a 96 well-microtitre plate as described previously (Brudzynski and Sjaarda, 2014). Each well-contained 50 μl of inoculum and 8 μl of twofold serial dilutions of glycoproteins (starting from 40 μg/ml). Bacterial growth was measured at an optical density at A595 nm using the Synergy HT multidetection microplate reader (Synergy HT, Bio-Tek Instruments, Winooski, VT, USA). The MIC was evaluated by recording the concentration of glycoproteins that reduced bacterial growth by 99% in comparison to a control, untreated culture, after 18 h incubation with shaking at 37°C. Statistical analysis and dose response curves were obtained using K4 software provided by Synergy HT (Bio-Tek Instruments, Winooski, VT, USA).

### Strains Tested

Strains were fresh clinical isolates from the Clinical Microbiology Laboratory, LHSC, London, ON, Canada. List of strains and their antibiotics susceptibility is presented in **Table 1**. The isolates tested per species were as follows: isolates of *E. coli*, three isolates of MRSA, three isolates of *P. aeruginosa*, four isolates of *K. pneumoniae*, four isolates of ESBL organisms including *Klebsiella oxytoca*, one isolates of *P. mirabilis*, one isolates of *E. coli* NDM-1, four isolates of VRE.

**Table 1:** Summary of MASCOT search results.

Protein band	Accession	Score	Expect	Peptide matches	Mass kDa	Name
61 kDa	gi| 58585098	281	2.6e-21	41	49311	MRJP1
	gi| 58585098	174	1.3e-10	22	49311	MRJP1
	gi| 58585108	149	4.1e-08	19	51441	MRJP2
29 kDa	gi| 58585108	325	8.6e+02	6 (2)	51441	MRJP2
	gi| 58585098	202	1.7e+02	4(1)	49311	MRJP1

Control strains were *E. coli* ATCC 14948 and *B. subtilis* ATCC 6633.

## Results

### MALDI TOF of Honey Glycoproteins

Honey glycoproteins used in this study were isolated using Concavalin A-agarose which binds high-mannose-type *N*-glycans with high affinity. The method allowed separation of glycoproteins carrying such structures from a bulk of honey proteins (Brudzynski and Sjaarda, 2015). The purity of glycoprotein fractions was further analyzed using one- and two-dimensional (2D) gel electrophoresis coupled with mass spectrometry.

**FIGURE 1:**
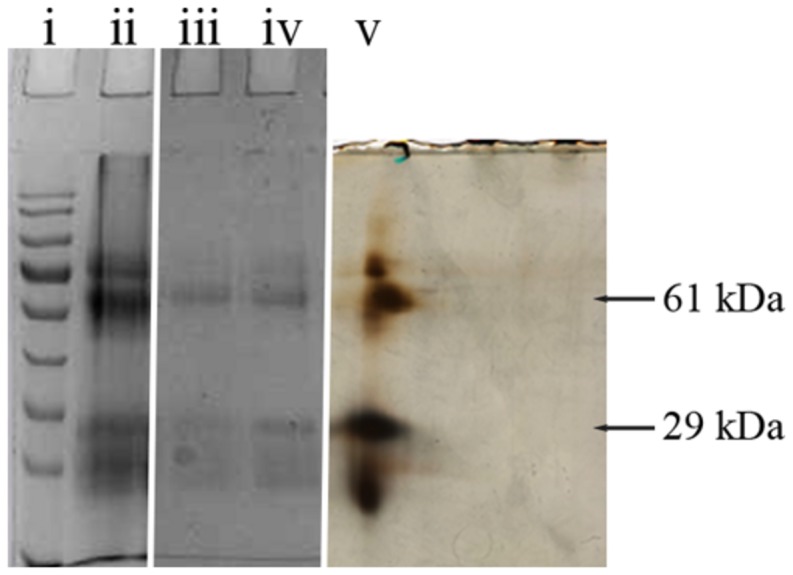
**1D and 2D gel electrophoresis of glycoprotein G208.** Lane “i” – protein molecular weight standards, “ii” – buckwheat honey H208, “iii” – glycoprotein G208, “iv” – glycoprotein G217, “v” – 2D gel electrophoresis of G208. The arrows indicate location of 61 and 29 kDa bands. The line over 2D represents pH gradient from pH 3 (left) to pH 10 (right).

Two selected spots from the main 61 and 29 kDa bands (**Figure 1**) were manually excised, subjected to in-gel tryptic digestion and the peptide mass spectra were analyzed by MALDI TOF. The generated peptide mass fingerprints were search using Mascot search engine against the NCBInr protein database. The data obtained revealed that the 61 and 29 kDa of G208 protein (**Figure 1**) presented a mixture of the major royal jelly proteins 1 and 2 precursors (*Apis mellifera*, accession number: gi 58585098- MRJP1 and gi 58585108-MRJP2). The obtained scores were 281, 174, and 149 for 61 kDa band and the scores of 325 and 202 for the 29 kDa band where the individual ions scores > 55 indicate identity or extensive homology (*p* < 0.05). The protein identification data is detailed in **Table 1**.

While both proteins, MRJP1 and MRJP2 are highly glycosylated (Kimura et al., 2006; Zhang et al., 2014), the important structural difference between MRJP1 and MRJP2 is the presence of antimicrobial peptides, Jelleins, in the C-terminal of MRJP1 molecule.

### Clinical Isolates

In order to investigate whether glps retain their antibacterial activities against MDR bacteria including ESBL, we used clinical isolates representing pathogens commonly involved in nosocomial infections. At their arrival, isolates obtained the lab numbers. The isolates included MRSA (*n* = 3), *P. aeruginosa* (*n* = 4), *K. pneumoniae* (*n* = 2), VRE (*n* = 2), and ESBL (*n* = 6; **Table 1**). After experiments were performed, the isolates were identified to genus and species and their susceptibility to antibiotics was confirmed using an automated system (Vitek 2^®^, Biomérieux^®^) by the Clinical Microbiology Laboratory, London Health Science Centre, London, ON, Canada.

**Table 2** indicates that microorganisms under study were simultaneously resistant to a number of antibiotics representing different chemical classes: β-lactams (piperacillin-tazobactam, ceftazidime, cefepime, ticarcillin-clavulanate), carbapenems (imipenem, meropenem), aminoglycosides (gentamicin, tobramycin, amikacin), and fluoroquinolones (ciprofloxacin). The isolates, therefore were classified as MDR pathogens. In addition, strains of *K. pneumonia* 1 and 2 (lab#1 and #19) and *E. coli* NDM 4 (lab #4) were recognized as possible carbapenemase producers and *K. pneumonia* 2 (lab #19) and ESBL-*P. mirabilis* showed resistance to colistin.

**Table 2:** Antibiogram of clinical isolates used in this study.

Antimicrobial	*Escherichia coli*				*Klebsiella pneumoniae* (*n* = 2)			*Pseudomonas aeruginosa*				*Proteus mirabilis*	Methicillin resistant MRSA			VRE *Enterococcus faecium*	
	1	2	3	4	1	2	3	1	2	3	4	1	1	2	3	1	2
	E	E	E	N			E					E					
	S	S	S	D			S					S					
	B	B	B	M			B					B					
	L	L	L				L					L					
Amikacin	S	S	S	R	R	R	S	S	S	R	R	S					
Amoxicillin-Clavulanate	R	R	R	R	R	R	R	R	R	R	R	S	R	R	R		
Ampicillin	R	R	R	R	R	R	R	R	R	R	R	R	R	R	R	R	R
Aztreonam	R	R	R	S	R	R	R	I	S	I	I	S					
Cefazolin	R	R	R	R	R	R	R	R	R	R	R	R	R	R	R	R	R
Cefepime	R	R	R	R	R	R	R	R	R	R	R	S					
Ceftazidime	R	R	R	R	R	R	R	R	R	R	R	S					
Cefoxitin	S	I	I	R	R	R	R	R	R	R	R	S	R	R	R	R	R
Ceftriaxone	R	R	R	R	R	R	R	R	R	R	R						
Cephalexin	R	R	R	R		R	R										
Chloramphenicol	S	S	S	S	R	R	S	R	R	R	R	S	S	S	S	S	S
Ciprofloxacin	R	R	R	R	R	R	S	R	R	R	R	S	R	R	R	R	R
Clindamycin													R	R	R	R	R
Colistin						R		S	S	S	S	R					
Daptomycin													S	S	S	S	S
Doxycyline													S	S	S	S	S
Ertapenem	S	S	S	R	R	R	R	R	R	R	R	S					
Erythromycin													R	R	R	R	R
Fosfomycin	S	S	S	S				R	R	R	R						
Fusidic acid																R	R
Gentamicin	S	S	S	R	R	R	R	R	R	R	R	R	S	S	S	R	R
Imipenem	S	S	S	R	R	S	S	R	R	R	R						
Linezoid													S	S	S	S	S
Meropenem	S	S	S	R	R	I	S	R	R	R	R	S					
Moxofloxacin													X	X	X		
Mupirocin high level													S	S	S		
Nitrofurantoin	S	S	S	R	R	R	R	R	R	R	R	R	S	S	S	R	R
Norfloxacin	S	R	R	R	R	R	S	R	R	R	R	S	R	R	R	R	R
Oxacillin													R	R	R		
Penicillin G													R	R	R	R	R
Piperacillin-tazobactam	R	S	I	R	R	R	R	R	R	S	R	S					
Pristinamycin																	
Quinupristin-dalfopristin													S	S	S	S	S
Rifampin													S	S	S		
Streptomycin-syn																R	R
Teicoplanin													S	S	S	R	R
Tetracycline	R	S	S	R	S	S	S	R	R	R	R	R					
Ticarcillin-Clavulanate	I	S	R	R	R	R	R	R	R	I	R	S					
Tigecycline	S	S	S	S	S	S	S					R					
Tobramycin	R	S	R	R	R	R	R	R	R	R	R	R					
Trimethoprim	R	S	R	R	R	R	S	R	R	R	R	S	S	S	S	R	R
Trimethoprim-Sulfamethoxazole	R	S	R	R	R	R	S	R	R	R	R	S	S	S	S	R	R
Vancomycin													S	S	S	R	R

### Susceptibility of Clinical Isolates to Glycoproteins

**FIGURE 2:**
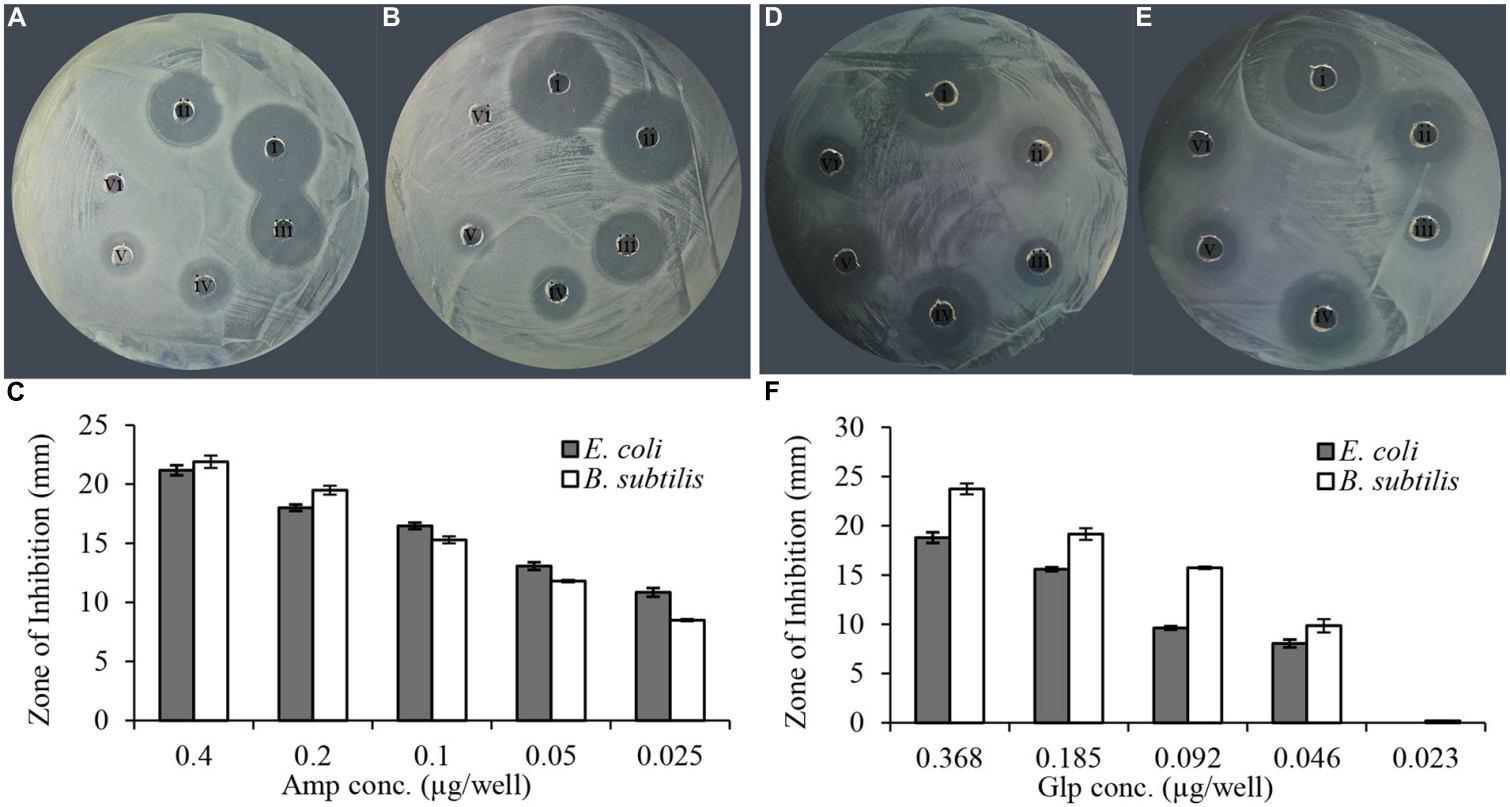
**Linear relationship between ampicillin and glycoprotein concentrations and the diameter of the zone of inhibition. (A)** Amp against *E. coli*, **(B)** Amp against *B. subtilis*, **(C)** relationship between the diameter of inhibition zone and ampicillin concentration and **(D)**. glp G208 against *E. coli*, **(E)** glp G208 against *B. subtilis*, and **(F)** relationship between the diameter of inhibition zone and glp concentrations. Columns represent means and standard errors.

*In vitro* evaluation of susceptibility of clinical isolates was performed using a semi-quantitative radial well-diffusion and broth microdilution assay. To compare the results of bacterial susceptibility to honey glycoproteins in a quantitative manner, the reference method has been designed that established the relationship between ampicillin dilutions and zone of inhibition (ZOI) in well-diffusion assay. As shown in **Figure 2**, the series of twofold diluted honey glycoproteins and ampicillin (1 mg/ml stock solutions) produced concentration-dependent ZOIs with R^2^ = 0.99, for ampicillin and R^2^ = 0.96 and 0.95 for glps tested against *E. coli* and *B. subtilis*, respectively. MIC values from well-diffusion assay against *E. coli* and *B. subtilis* (1 × 10^6^ CFU/ml) were <0.025 μg/well or <2.0 μg/ml for ampicillin and <0.046 μg/well or 5.7 μg/ml for glps.

**FIGURE 3:**
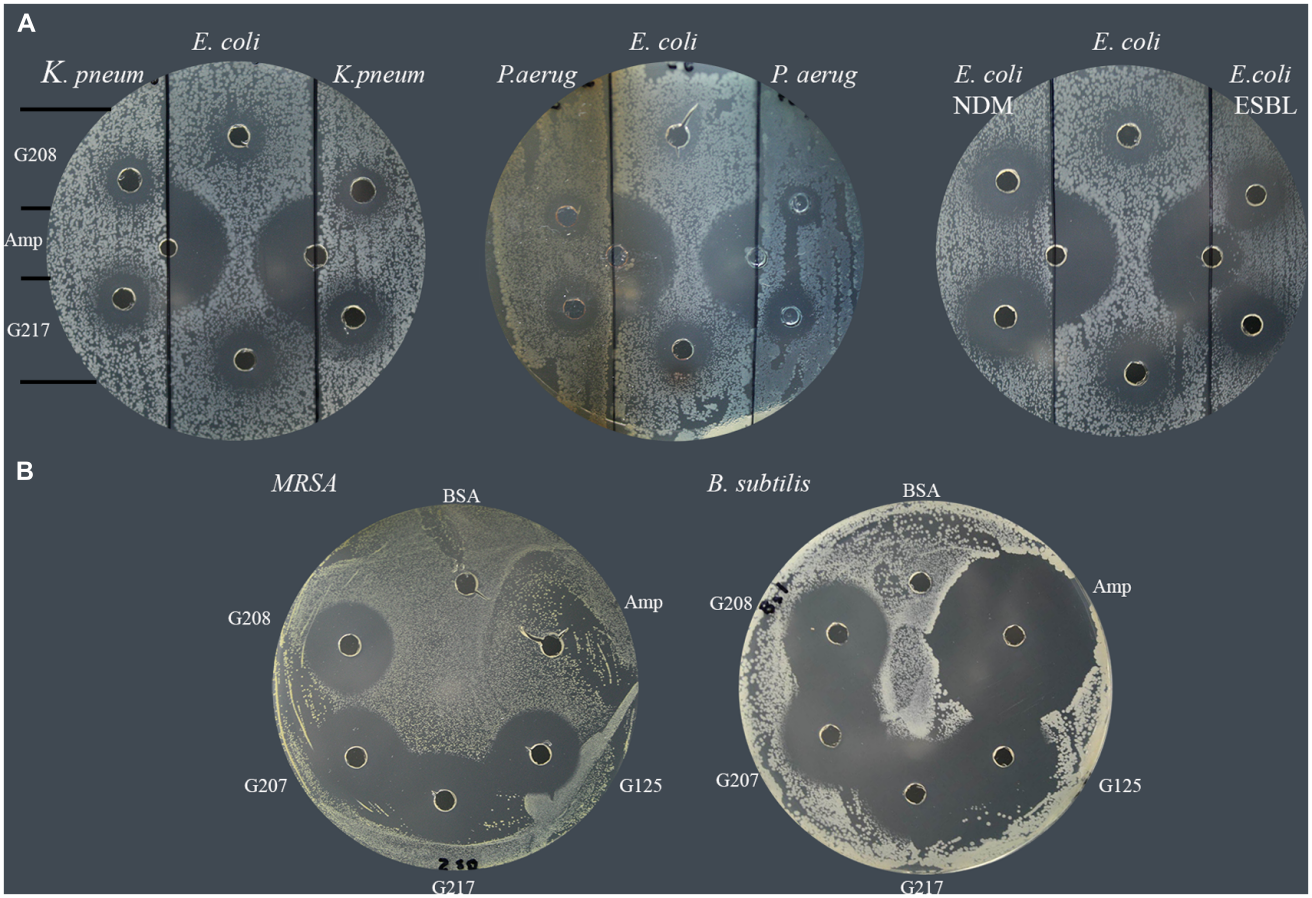
**Susceptibility of multidrug resistant clinical isolates to honey glycoproteins. (A)** Susceptibility of *K. pneumonia* (# 1and #19), *P. aeruginosa* (#34, and #35), *E. coli* NDM (#4) and *E. coli*. ESBL (# 7) to G208 and G217. Ampicillin-sensitive *E. coli* (ATCC14948) served as a control. **(B)** Susceptibility of MRSA 1 (#5) and ampicillin-sensitive *B. subtilis* (ATCC 6633) to G208, G207, G217 and G125 isolated from different honeys.

In a similar manner, each of isolates was tested for susceptibility to glycoproteins by well-diffusion assay. The agar plates were divided into three sections; the middle section was used to spread plating of the standard *E. coli* (ATCC 14948) sensitive to ampicillin while the right and left sections were used to spread plate with the MDR isolates. The susceptibility of isolates to two glycoproteins, G208 and G217, isolated from two different buckwheat honeys, was then evaluated and compared to that of ampicillin-sensitive control. Both glycoproteins have been shown to exhibit antibacterial activity against *K. pneumonia* 1 and 2 (**Table 2**; lab number #1 and #19), *P. aeruginosa* 3 and 4 (lab number #34 and #35), *E. coli* NDM 4 (lab number #4), and *E. coli* ESBL 2 (lab number #7). These results indicate that MDR of isolates to antibiotics did not affect antibacterial action of glycoproteins (**Figure 3** and **Table 2**).

All MRSA isolates were also highly sensitive to honey glycoproteins (**Figure 3**). Moreover, in addition to G208, glycoproteins G217, G125, and G207 isolated from different honeys, produced zones of inhibition of comparable diameters (**Figures 2** and **3**), indicating that antibacterial activity of glycoproteins resides in their common structure.

**FIGURE 4:**
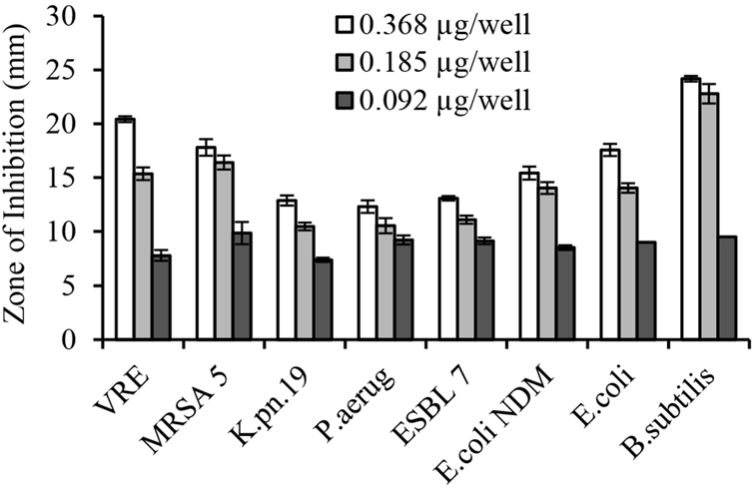
**Concentration-dependent growth inhibition of multi-drug resistant clinical isolates by honey glycoproteins in well-diffusion assay**.

The serial two-fold dilution of glps showed a concentration-dependent inhibition of growth of isolates in the radial diffusion assay with susceptibility <0.1 μg/well or <12.5 μg/ml (**Figure 4**). Differences in the mean of inhibition zone diameters between bacterial species pointed out to some variation in the sensitivity of individual strain to glps. Generally, glps were found less potent in inhibition of growth of *K. pneumonia* and *P. aeruginosa* compared to MRSA, VRE and *E. coli* NDM or standard, antibiotic-sensitive bacteria, as judged by the size of ZOI they produced at comparable concentrations (**Figures 3** and **4**).

**FIGURE 5:**
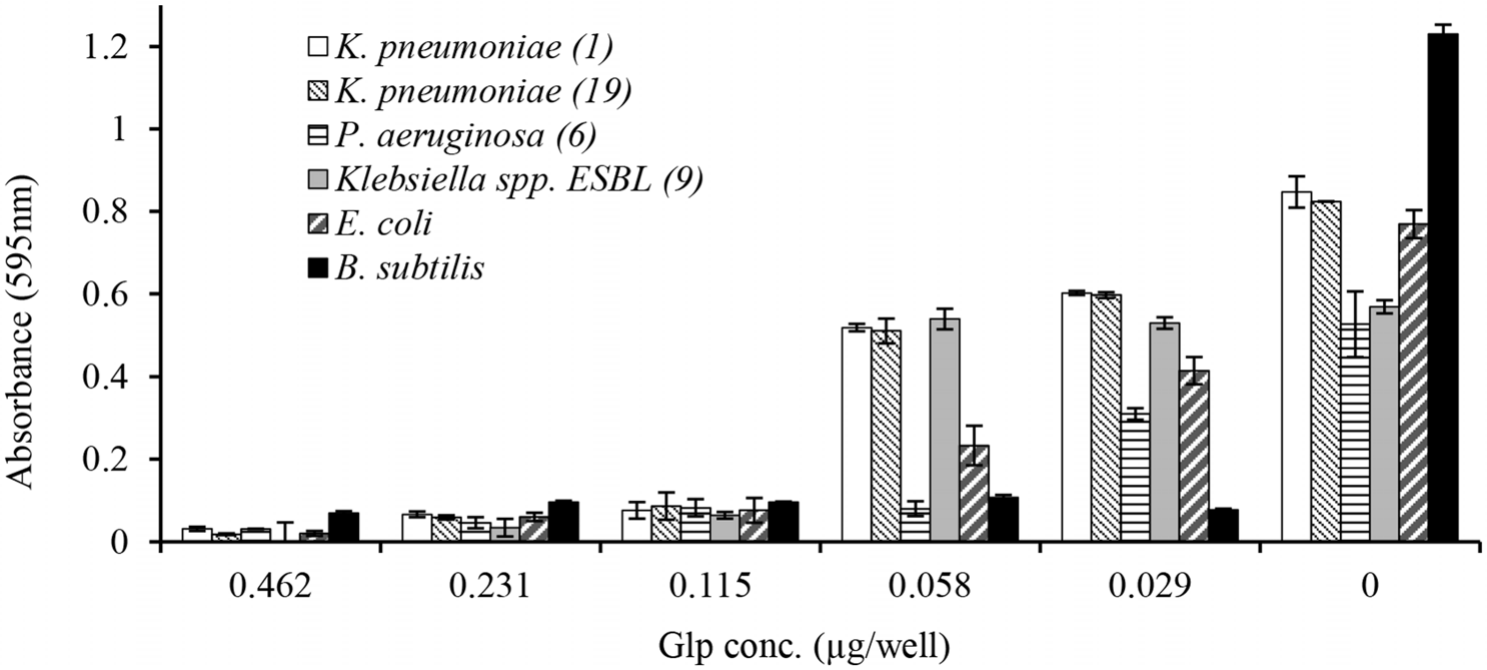
**Concentration-dependent growth inhibition of clinical isolates by honey glycoproteins evaluated using broth microdilution assay**.

In broth microdiltuion assay, glps showed considerable antibacterial activity with MIC_90_ values ranging from 4.8 μg/ml against *B. subtilis* to 14.4 μg/ml against *K. pneumoniae, Klebsiella* spp. ESBL(lab number #9) and *E. coli* (**Figure 5** and **Table 3**). However, *P. aeruginosa* susceptibility differed between individual strains ranging from 5.7 to 33 μg/ml for *P. aeruginosa* (#6) and *P. aeruginosa* #35, respectively.

**Table 3:** Minimum inhibitory concentrations (MIC).

Bacterial strain	MIC_90_ (μg/ml)	Antibiogram (**Table 2**)
MRSA (#5)	5.4	MRSA No. 1
MRSA (#6)	5.4	MRSA No.2
*Klebsiella pneumonia* (#1)	14.4	*K. pneumonia* No.1
*K. pneumonia* (#19)	14.4	*K. pneumonia* No.2
*Pseudomonas aeruginosa* (#34)	25.5	*P. aeruginosa* No.3
*P. aeruginosa* (#35)	33	*P. aeruginosa* No.4
*P. aeruginosa* (#6)	5.7	*P. aeruginosa* No.1
ESBL *P. mirabilis* (#2)	5.7	*P. mirabilis* No.1
ESBL *Escherichia coli* NDM (#4)	5.7	*E. coli* No.4
ESBL *E. coli* (#7)	23	*E. coli* No.1
ESBL *E. coli* (#8)	14.4	*E. coli* No.2
ESBL *Klebsiella* spp. (#9)	14.4	*K. pneumonia* No.3
ESBL *E. coli* (#10)	24.0	*E. coli* No.3
VRE (#17)	5.4	VRE No.1
VRE	5.7	VRE No.2
*E. coli* ATCC	15.8	
*B. subtilis* ATCC	4.8	

Thus, honey glps showed activity against MDR clinical isolates, irrespectively of the mechanism by which the resistance was conferred.

## Discussion

This study revealed that honey glycoproteins containing Major Royal Jelly Proteins 1 and 2 possess a broad-spectrum of antimicrobial activity against several clinical isolates of MDR including ESBL producing gram-negative bacteria. Recently, we have shown that glps are the membrane active molecules; the treatment of bacterial cells with glps increased membrane permeability for propidium iodide in 90% cells as indicated by flow cytometry, caused release of endotoxins from *E. coli*, changed the cell shape of treated bacteria with formation of filaments and spheroplasts, and finally evoked cell lysis as evidenced by light and scanning electron microscopy (Brudzynski and Sjaarda, 2014, 2015). Both MRJP1 and 2 are highly glycosylated proteins with the high-mannose glycans attached to the protein backbone ranging from 28 up to 178 glycosylation sites per molecule, respectively (Zhang et al., 2014). Due to the presence of carbohydrate moiety, glps displayed lectin-like activity, agglutinating both Gram-positive *B. subtilis* and Gram-negative *E. coli.* Importantly, glps were able to efficiently lyse bacteria. Bacterial killing by glps occurred very rapidly after addition of glps to the bacterial cultures (Brudzynski and Sjaarda, 2015). The most important structural difference between the MRJP1 and 2 is the presence of three antimicrobial peptides at the MRJP1 C-terminus (Fontana et al., 2004). As it was impossible to separate MRJP1- from MRJP2- containing glycoproteins in this study, we used their natural mixture to gain a better understanding whether the antibacterial effects of glps would be retain against pathogens that develop several resistance mechanisms.

An antibiogram of clinical isolates used in this study indicates that in addition to being resistant to β-lactams, these isolates were also resistant to carbapenems, fluoroquinolones, aminoglycosides and tetracyclines, therefore presenting MDR phenotype. Among isolates tested, there were (ESBL)-producing carbapenem-resistant *K. pneumoniae* (KPC), *P. aeruginosa* (resistant to imipenem and cephalosporin, ceftazidime) and *E. coli* NDM-1(New Delhi metallo-β-lactamase).

Despite ESBL and the coexistence of multiple resistance genes, the isolates were susceptible to glps action. Both, well-diffusion assay and broth microdilution assay provided evidence that glps inhibited growth of MDR in a concentration-dependent manner. The most sensitive isolates comprised MRSA and VRE with MIC_90_ values of 5.4–5.7 μg/ml followed by two strains of carbapenemase-producing *K. pneumonia* with MIC 14.4 μg/ml. All ESBL tested were inhibited by glps with MIC ranging from 5.7 μg/ml against *P. mirabilis* and *E. coli* NDM to 14.4 μg/ml against ESBL *E. coli* 14.4. However, highly resistant strains of *P. aeruginosa* (#34 and#35) and one strain of ESBL *E. coli* (#10) were the least sensitive to glps, with MICs in the range of 33 and 24 μg/ml, respectively. Thus, glps exhibited a consistent activity against the MDR isolates irrespectively of the resistance mechanism. This indicates that glps must exert their antibacterial action by yet a different mode of action than other classes of β-lactams. The fact that glycoproteins G217, G125, and G207 isolated from different honey varieties also efficiently inhibited growth of MDR points to the glps structure as the lead structure responsible for the activity. Moreover, glps activity against MDR was concentration-dependent as shown in both a semi-quantitative well-diffusion and broth microdilution assays, providing further evidence for the structure-activity relationship. Despite their considerable size (61 kDa), glps exhibited antibacterial activity at the microgram/ml concentrations, being in a range with MICs of other classes of β-lactams. Our ongoing study on the structure-activity relationship in which the antibacterial activity of full-length glycoprotein G208 was compared to that of synthetic Jelleins, (the antimicrobial peptides present in the G208 molecule) showed that both compounds have a broad spectrum of activity against Gram-positive and Gram-negative bacteria and rapidly kill bacteria at very low concentrations (paper submitted). Therefore, the bactericidal activity of glps may result, at least in part, from the action of the antimicrobial peptides, Jelleins.

These results show that glps structure is the lead structure carrying glps antibacterial activity. Thus, honey MRJP-containing glycoproteins possess suitable characteristics to be considered a novel antibacterial drug candidate.

## Conflict of Interest Statement

The authors declare that the research was conducted in the absence of any commercial or financial relationships that could be construed as a potential conflict of interest.
